# P-1884. Online Medical Education Effectively Improves Clinician Knowledge, Competence and Confidence in Implementing Antimicrobial Stewardship Practices When Diagnosing and Managing Suspected Gastrointestinal Infections with Available Molecular Rapid Diagnostic Tests

**DOI:** 10.1093/ofid/ofaf695.2053

**Published:** 2026-01-11

**Authors:** Arun S Nair, Roderick Smith, James Martorano

**Affiliations:** Medscape Education, TARRYTOWN, New York; Medscape, New York, New York; Medscape EDucation, New York, NY

## Abstract

**Background:**

This study evaluated the impact of online CME on knowledge, competence, and confidence in integrating molecular rapid diagnostic tests (mRDTs) and antimicrobial stewardship (AMS) strategies for diagnosing and managing suspected gastrointestinal (GI) infections.

**Methods:**

We developed an on-demand 30-minute online CME activity presented by three experts with accompanying slides. Educational impact were assessed with learners completing pre-/post-assessment questions using a matched pre-/post-assessment design. A paired samples t-test measured significance in overall correct responses and confidence ratings, while a McNemar test assessed question-level changes (*P* < .05). Confidence was rated on a 5-point Likert scale. Data were collected from 8/2024 to 4/2025.

**Results:**

As of 4/2025, the curriculum reached ∼24,000 learners, primarily Gastroenterologists (Gastros), Primary Care Physicians (PCPs), and Infectious Disease (ID) Specialists. Data from pre-/post-assessments showed significant learning improvements (*P* < .001). Overall, 28% of Gastros (n=226), 32% of PCPs (n=275), and 12% of ID Specialists (n=191) improved knowledge, competence, and/or confidence in implementing mRDTs and AMS practices for suspected GI infections.

Learner specific insights from the assessment include:

o ID Specialists had higher baseline knowledge, with pre-education scores averaging 84%, reflecting familiarity with mRDTs. Gastros and PCPs had lower baseline scores, averaging 70% and 63%, respectively

o In a case assessment on managing suspected GI infections with mRDTs, 23% of Gastros and 30% of PCPs initially chose broad-spectrum antibiotics before identifying the causative agent, highlighting gaps in AMS practices. Post-education, inappropriate antibiotic use among Gastros and PCPs decreased by ∼20%, demonstrating the program's effectiveness in promoting evidence-based practices

**Conclusion:**

These findings show that online CME/CE supports AMS adoption for managing suspected GI infections with mRDTs. Future education should enhance mRDT interpretation among Gastros and PCPs, address AMS gaps, and provide case-based learning to reinforce applications of these diagnostic tools.

**Disclosures:**

All Authors: No reported disclosures

